# Middle Ear Salivary Choristoma: A Rare Case Report and Update on Congenital Associations, Facial Nerve Involvement, and Treatment Strategies

**DOI:** 10.1155/2020/8435140

**Published:** 2020-08-24

**Authors:** Allen Young, Lauran Evans, Matthew Ng

**Affiliations:** ^1^Department of Otolaryngology Head and Neck Surgery, University of Nevada Las Vegas, 1701 West Charleston Boulevard Suite 490, Las Vegas, NV 89102, USA; ^2^Department of Head and Neck Surgery, University of California Los Angeles, 10833 Le Conte Avenue CHS 62-132, Los Angeles, CA 90095, USA

## Abstract

Salivary gland choristoma is an extremely rare middle ear mass and is hypothesized to be caused by second branchial arch developmental anomalies. We present a 14-year-old girl with Dandy–Walker syndrome and conductive hearing loss. Middle ear exploration revealed a large middle ear mass with absent incus and stapes and displaced facial nerve. The mass was completely excised with histological confirmation of salivary gland choristoma. Her hearing was improved with bone-anchored hearing aids (BAHA). As facial nerve involvement is common, physicians should consider partial excision to avoid facial nerve palsy. Hearing restoration can be achieved with OCR or BAHA.

## 1. Introduction

A choristoma is a congenital, benign overgrowth of mature tissue found in an abnormal location of the body. On extremely rare occasions, benign salivary gland tissue can be present in the middle ear cavity. Presenting symptoms include conductive hearing loss, ear fullness, tinnitus, otalgia, and otorrhea [1, 2]. These salivary gland choristomas are also associated with malformations of the ossicular chain, particularly the incus and stapes, along with dehiscence of the facial nerve from the fallopian canal [2, 3]. Their rare presentation and proximity to vital structures of the middle ear make diagnosis and treatment a challenge. We present a case report detailing this rare presentation along with an extensive review of previous cases with associated congenital findings, facial nerve involvement and its impact on surgery, and hearing augmentation outcomes.

## 2. Methods

We present a unique clinical case from our institution that is in line with the Surgical Case Report (SCARE) criteria along with a thorough literature review of all salivary gland choristomas, focusing on congenital and middle ear malformations, facial nerve involvement, treatments, and outcomes [4]. Statistical analysis was performed with Fisher's exact test using Statistical Analysis Software (SAS) version 9.4.

## 3. Case Report

**Figure 1:**
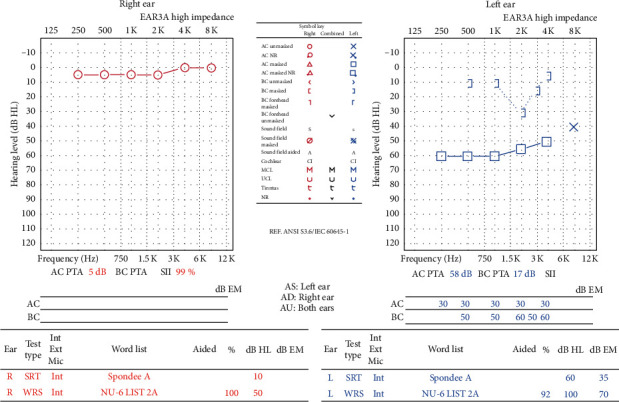
Audiogram showing normal hearing on the right and a moderate-severe rising to moderate mixed hearing loss with 25–50 dB air-bone gap on the left. Word recognition scores were excellent bilaterally.

**Figure 2:**
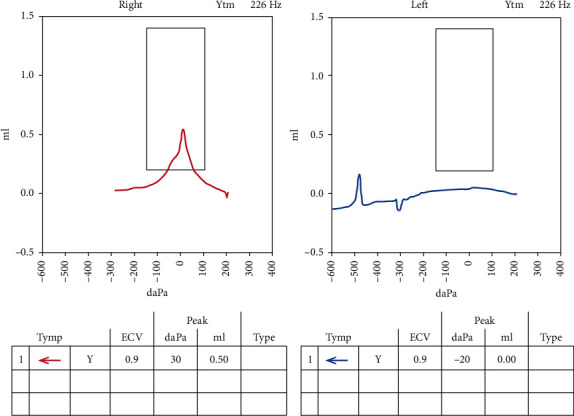
Tympanogram showing type A for the right ear and type B for the left ear.

**Figure 3:**
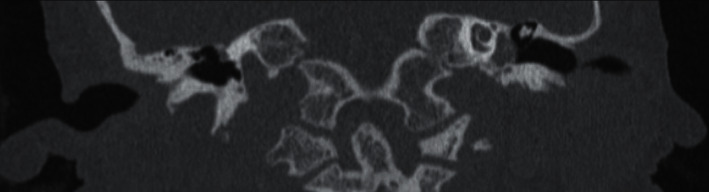
CT coronal view. Left mesotympanic and hypotympanic mass extending into the infracochlear space. Visible sharp.

**Figure 4:**
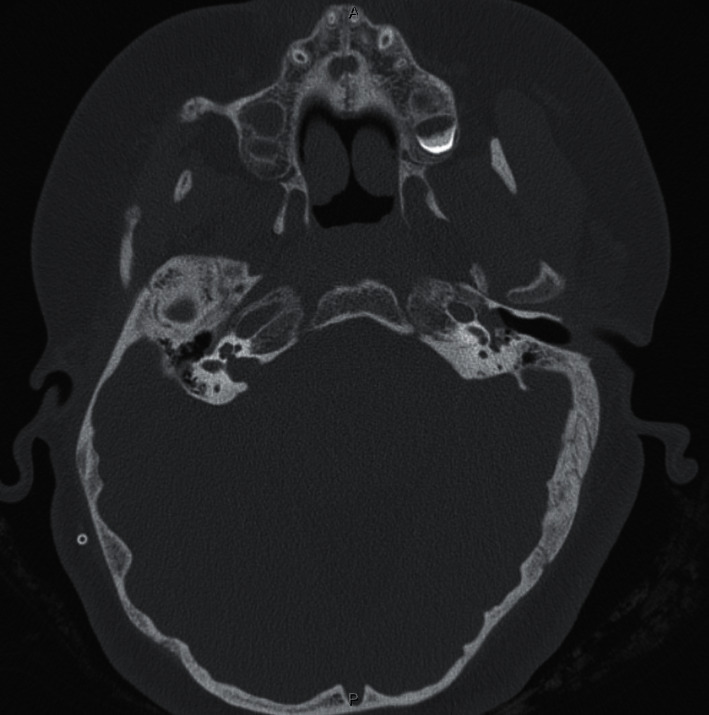
CT axial view. There is no visible stapes. There is no direct continuity between the mass and the carotid or jugular bulb.

A 14-year-old female with a history of Dandy–Walker syndrome, hydrocephalus with ventriculoperitoneal shunt placement, and recurrent otitis media presented to our academic center with left hearing loss and aural fullness. Tympanostomy tubes were placed in each ear 5 years prior. Her mother states the hearing loss was present since birth and denies any otorrhea, otalgia, aural bleeding, dizziness, vertigo, or familial hearing loss. Audiogram showed normal hearing on the right and a moderate-severe rising to moderate mixed hearing loss with 25–50 dB air-bone gap on the left. Word recognition scores were excellent bilaterally (Figure 1). Tympanogram was type A for the right and type B for the left (Figure 2). CT temporal bone showed a left mesotympanic and hypotympanic mass extending into the infracochlear space. The scutum was sharp. There was no visible stapes. There was no direct continuity between the mass and the carotid or jugular bulb (Figures 3 and 4).

On examination, the left tympanic membrane had a shallow retraction pocket in the pars flaccida and a white retrotympanic mass suspicious for a possible secondary-acquired cholesteatoma. The decision was made to perform an exploratory tympanotomy and removal of the mass.

Under continuous facial nerve monitoring, tympanomeatal flap was elevated by a senior otology surgeon who uncovered a white, smooth, and mildly pulsatile mass. Compression of the external jugular veins and increased vagal pressure did not expand the mass. Mild compression of the mass did not cause blanching. Electrical stimulation of the mass at the inferior pole of the tumor and marching superiorly showed no electromyography (EMG) response. Bone removal from the canal floor was required to gain better access to the tumor. The tumor was first mobilized from its infracochlear position followed by the superior pole using curved dissectors. Total tumor removal was possible without any facial nerve stimulation.

After complete tumor removal, middle ear inspection revealed malformed long process of the incus without a stapes superstructure or oval window niche for reconstruction. An inferiorly displaced tympanic segment of the facial nerve was identified and was able to be electrically stimulated.

Pathology showed a 1 cm polypoid mass of respiratory sinus mucosa lined with pseudostratified columnar ciliated epithelium and scattered goblet cells, consistent with a salivary gland choristoma of the middle ear. There was no dysplasia or malignancy identified. On 2-week postoperative visit, she had no facial nerve weakness, and her aural fullness had improved, much to her satisfaction. Although ossicular chain reconstruction was not possible intraoperatively due to nonvisualized oval niche, the patient showed promising hearing response to a bone-anchored hearing aid (BAHA).

## 4. Discussion

Salivary gland choristoma in the middle ear cavity is an extremely rare condition with fewer than 50 cases ever reported [1, 2]. There appears to be a left-sided and female predominance, with age ranging from 9 months to 52 years old [5].

Salivary gland choristoma of the middle ear is hypothesized to form from malformation of the second branchial arch prior to the fourth month of gestation [4, 6]. Salivary tissue becomes trapped in the middle ear during the fusion of the tympanic ring with the temporal bone [7]. Abnormal second branchial arch development may also underpin the frequent findings of malformed incus, stapes, and facial nerve canal [7].

Although the abnormal deposition of tissue is often isolated to the middle ear, a unique developmental syndrome may be present [5, 6]. Several ipsilateral auricle and facial abnormalities have been associated with salivary choristomas, including alopecia [4, 8], auricle deformities and swelling [9], preauricular fistulas [10], and facial asymmetry [6]. Dandy–Walker syndrome is a congenital hypoplasia of the cerebellar vermis, dilation of the fourth ventricle, and enlarged posterior fossa. To date, this syndrome has not been known to be related to any choristomas. However, Buckmiller et al. described a case of an encephalocele in a child with a contralateral middle ear salivary choristoma [5] (Table 1).

CT imaging and otomicroscopic appearance are usually sufficient to presumptively diagnose middle ear pathology before making the first incision. However, due to the rarity of middle ear salivary choristoma, it is often misdiagnosed as cholesteatoma given its white, retrotympanic appearance [37] or otosclerosis due to the unilateral conductive hearing loss [8, 38]. Only one case had concurrent cholesteatomas with a middle ear salivary choristoma [35]. Regardless of initial diagnosis, exploratory tympanotomy is warranted in all cases. Intracranial and vascular tumors can be ruled out through a preoperative CT scan, enlarging mass during increased intracranial pressure through Valsalva or occlusion of internal jugular veins (Queckenstedt maneuver), and blanching of mass during manipulation. Histopathology of the middle ear mass is required for diagnosis confirmation.

Malignant transformation of salivary choristoma is rare [16], and many authors in the literature suggest that complete tumor excision is not necessary [10, 44]. However, others propose that the small chance of malignant transformation does warrant complete excision [45].

The major limiting factor for complete surgical excision is facial nerve involvement. Cases involving the facial nerve had a significantly lower complete excision rate than those in which the facial nerve was spared (44.4% vs. 84.2%, *p*=0.0027, Table 2). Permanent facial nerve palsy after removal of a middle ear salivary choristoma has occurred in two reported cases [36, 46], with two additional cases of transient facial nerve palsy that later recovered [38, 47]. To mitigate this risk of facial nerve palsy in all cases, use of a facial nerve monitor during surgery is strongly recommended [4, 44]. The abnormal and dehiscent course of the facial nerve makes it vulnerable to injury during middle ear dissection. Facial nerve electrical stimulation should be applied to the mass at areas prior to surgical manipulation to ensure the facial nerve is not in close proximity, and electrocautery of the mass should be avoided. Partial excision of mass can be considered to avoid facial nerve palsy.

Ossicular chain reconstruction has been attempted both during initial removal [45] and staged months after removal [37] for restoration of hearing, with promising results (Table 3). Hearing was significantly improved in patients who underwent OCR than those without repair (58.3% vs. 12.9%, *p*=0.0047). In our present case, OCR was not attempted due to inaccessibility of the oval window niche. However, bone-anchored hearing aids (BAHA) can provide a viable alternative to hearing enhancement as our patient showed encouraging response on BAHA demo.

## 5. Conclusion

Salivary gland choristoma is an extremely rare diagnosis of a middle ear mass. Radiographic imaging and careful middle ear exploration are needed to rule out vascular and intracranial tumors. As facial nerve involvement is common, physicians may consider partial excision to avoid facial nerve palsy. Hearing restoration can be achieved with ossicular chain reconstruction or BAHA.

## Figures and Tables

**Table 1:** Review of middle ear salivary choristomas and characteristics.

Authors	Year	Age	Sex	Laterality	Congenital defects	Middle ear malformations	Facial nerve	Surgical excision vs. biopsy	Hearing augmentation repair	Postoperative findings
Taylor et al. [11]	1961	31	F	L	None	Incus, stapes	Y	Biopsy	No	Hearing unchanged
Steffen et al. [12]	1962	52	F	R	None	Incus, stapes		Biopsy	No	Hearing unchanged
Noguera et al. [13]	1964	19	M	L	None	Incus, stapes		Biopsy	OCR	Hearing unchanged
Caplinger et al. [14]	1967	34	F	L	Auricle swelling	Malleus, incus		Excision	No	Hearing unchanged
Bruner et al. [15]	1970	6	F	R	None	Stapes	Y	Excision	OCR	Hearing improved, transient facial palsy
Hociota et al.	1975	50	M	L	None	Stapes	Y	Excision	No	Facial paralysis
Peron et al. [16]	1975	20	M	Bilateral	Developmental delay, bilateral cholesteatomas	Malleus, incus, stapes		Autopsy	No	
Mischke et al. [17]	1977	9	F	L	Postauricular cystic mass	Malleus, incus	Y	Biopsy	No	.
Wine et al. [18]	1977	20	M	L	None		Y	Biopsy	No	Hearing improved
Abadir et al. [7]	1978	21	F	L	Microtia	Incus, stapes	Y	Excision	OCR	Hearing unchanged
Kley et al. [19]	1979	46	F	R	None		Y	Excision	Yes	Hearing improved
Cannon et al. [10]	1980	24	F	R	Auricle swelling	Incus, stapes	N	Excision	No	
Quaranta et al. [9]	1981	23	M	L	Alopecia	Incus		Excision	No	.
Saeger et al. [20]	1982	17	M	L	None	Incus	Y	Biopsy	No	.
Saeger et al. [20]	1982	10	F	L	None	Malleus, incus	N	Excision	Yes	.
Moore et al. [21]	1984	5	F	L	None	Incus, stapes		Excision	No	
Kartush et al. [22]	1984	19	F	R	Auricle swelling	Incus		Biopsy	No	Hearing unchanged
Bottrill et al. [23]	1992	10	M	R	None	Incus, stapes.	N	Excision	No	Hearing improved
Cejas-Mendez et al. [24]	1992	3	F	L	EAC malformation	.	N	Excision	No	Hearing unchanged
Munster et al. [25]	1994	16	M	R	None	Incus, stapes	N	Biopsy	No	.
Namdar et al. [5]	1995	6	F	L	EAC malformation	Incus, stapes	Y	Biopsy	No	Hearing unchanged
Hinni et al. [26]	1996	9	M	L	None	Malleus, incus, stapes	N	Excision	No	.
Anderhuber et al. [27]	1996	4	M	L	None	Incus, stapes	N	Excision	No	Hearing unchanged
Perry et al. [28]	1998	5	M	L	Branchial cleft cyst	Incus	Y	Biopsy	No	
Morimoto et al. [3]	1999	13	M	L	Alopecia	Incus, stapes	Y	Excision	No	
Supiyaphun et al. [29]	2000	10	F	L	EAC malformation	Malleus, incus, stapes	Y	Excision	No	Hearing unchanged
Ha et al. [30]	2000	3	F	R	None	None	N	Excision	No	Hearing unchanged
Vasama et al. [8]	2001	.	.	R	.	Incus	Y	.	.	.
Buckmiller et al. [6]	2001	0.75	M	R	Facial asymmetry, bilateral preauricular pits, encephalocele, Mondini-type deformities	Incus, stapes		Excision	No	.
Ookuchi et al.	2003	1	F	R	None	Incus, stapes	Y	Biopsy	No	
Simoni et al. [31]	2003	0.83	F	L	Tonsillar teratoid polyp	None	N	Excision	No	Hearing unchanged
Enoz et al. [32]	2006	14	F	L	None	Stapes	Y	Biopsy	No	
Yazici et al.	2006	32	F	L	None	Stapes	N	Excision	No	Hearing unchanged
Lee et al. [33]	2006	0.9166	F	R	None	Incus, stapes	N	Excision	OCR	Hearing improved
Boleas-Aguirre et al. [34]	2006	12	M	L	.	Incus, stapes	N	.	.	.
Nassar et al. [35]	2007	32	M	R	None	Incus, stapes	Y	Excision	OCR	Hearing improvement, transient facial nerve palsy
Toros et al. [36]	2010	7	F	R	Situs inversus	Incus	N	Excision	OCR	Hearing improved
Gomez et al.	2013	32	F	L	None	Normal		Biopsy	No	Facial nerve palsy
Amrhein et al. [37]	2014	0.83	F	R	Branchio-oto-renal syndrome. Developmental delay, bilateral preauricular pits, ear tag, dysplastic auricle	Malleus, incus, stapes		Excision	OCR, BAHA	Hearing unchanged
Fois et al. [38]	2014	22	F	L	None	Incus, stapes	N	Excision	OCR	Hearing improved
Chen et al. [39]	2015	6	F	L	Preauricular pit		Y	Biopsy	No	Hearing unchanged
Noda et al. [40]	2016	10	F	L	Alopecia	Stapes	Y	Biopsy	OCR	Hearing improved
Aghazadeh et al. [1]	2016	41	M	R	None	Stapes	N	Excision	No	Hearing improved
Ziari et al. [41]	2016	39	M	L	None	Stapes	N	Excision	No	Hearing improved
Su et al. [42]	2019	8	F	L	Pharyngeal hamartoma	Malleus, incus	N	Excision	Yes	Hearing unchanged
Purnell et al. [43]	2019	6	M	L	Ear tag	Incus	N	Excision	OCR	
Current case	2019	14	F	L	Dandy–Walker	Incus, stapes	N	Excision	BAHA	Hearing improved

F: female, M: male, R: right, L: left, Y: yes, and N: no. All unfilled boxes indicate information that was unspecified in the case reports.

**Table 2:** Facial nerve involvement.

Facial nerve	Total	Complete excision	Biopsy/partial excision	*p* value
Involved	18	8 (44.4%)	10 (55.6%)	0.0027
Not involved	19	16 (84.2%)	1 (5.2%)	—

Complete excision vs. biopsy/partial excision has different risks to the facial nerve. Two cases that had no facial nerve involvement and did not specify the exact procedure may underestimate the risk of facial nerve involvement during complete excision.

**Table 3:** Hearing repair outcomes.

Hearing repair	Total	Hearing improvement	No improvement or no follow-up recorded	*p* value
Ossicular chain reconstruction (OCR)	12	7 (58.3%)	5 (41.7%)	0.0047
No repair	31	4 (12.9%)	27 (87.1%)	—

## References

[1] Aghazadeh K., Karimi E., Sharifi A. (2016). Salivary gland choristoma of the middle ear: a case report. *Case Reports in Clinical Practice*.

[2] Rinaldo A., Ferlito A., Devaney K. O. (2004). Salivary gland choristoma of the middle ear. *Orl*.

[3] Morimoto N., Ogawa K., Kanzaki J. (1999). Salivary gland choristoma in the middle ear: a case report. *American Journal of Otolaryngology*.

[4] Agha R. A., Borrelli M. R., Farwana R. (2018). The SCARE 2018 statement: updating consensus surgical CAse REport (SCARE) guidelines. *International Journal of Surgery*.

[5] Namdar I., Smouha E. E., Kane P. (1995). Salivary gland choristoma of the middle ear: role of intraoperative facial nerve monitoring. *Otolaryngology-Head and Neck Surgery*.

[6] Buckmiller L. M., Brodie H. A., Doyle K. J., Nemzek W. (2001). Choristoma of the middle ear: a component of a new syndrome?. *Otology & Neurotology*.

[7] Abadir W. F., Pease W. S. (1978). Salivary gland choristoma of the middle ear. *The Journal of Laryngology & Otology*.

[8] Vasama J.-P., Ramsay H., Markkola A. (2001). Choristoma of the middle ear. *Otology & Neurotology*.

[9] Quaranta A., Mininni F., Rwsta L. (1981). Salivary gland choristoma of the middle ear: a case report. *The Journal of Laryngology & Otology*.

[10] Cannon C. R. (1980). Salivary gland choristoma of the middle ear. *The American Journal of Otology*.

[11] Taylor G. D., Martin H. F. (1961). Salivary gland tissue in the middle ear: a rare tumor. *Archives of Otolaryngology—Head and Neck Surgery*.

[12] Steffen T. N., House W. F. (1962). Salivary gland choristoma of the middle ear. *Archives of Otolaryngology—Head and Neck Surgery*.

[13] Noguera J. T., Haase F. R. (1964). Congenital ossicular defects with a normal auditory canal: its surgical treatment. *Eye, Ear, Nose & Throat Monthly*.

[14] Caplinger C. B., Hora J. F. (1967). Middle ear choristoma with absent oval window: a report of one case. *Archives of Otolaryngology—Head and Neck Surgery*.

[15] Bruner R. C. (1970). Salivary gland choristoma of the middle ear: a case report. *Archives of Otolaryngology—Head and Neck Surgery*.

[16] Peron D. L., Schuknecht H. F. (1975). Congenital cholesteatomata with other anomalies. *Archives of Otolaryngology—Head and Neck Surgery*.

[17] Mischke R. E., Brackmann D. E., Gruskin P. (1977). Salivary gland choristoma of the middle ear. *Archives of Otolaryngology*.

[18] Wine C. J., Metcalf J. E. (1977). Salivary gland choristoma of the middle ear and mastoid. *Archives of Otolaryngology*.

[19] Kley H. A. (1979). Monomorphous tubular salivary gland adenoma of the middle ear (in German). *Laryngologie, Rhinologie, Otologie*.

[20] Saeger K. L., Gruskin P., Carberry J. N. (1982). Salivary gland choristoma of the middle ear. *Archives of Pathology & Laboratory Medicine*.

[21] Moore P. J., Benjamin B. N. P., Kan A. E., Kan A. (1984). Salivary gland choristoma of the middle ear. *International Journal of Pediatric Otorhinolaryngology*.

[22] Kartush J. M., Graham M. D. (1984). Salivary gland choristoma of the middle ear: a case report and review of the literature. *The Laryngoscope*.

[23] Bottrill I. D., Chawla O. P., Ramsay A. D. (1992). Salivary gland choristoma of the middle ear. *The Journal of Laryngology & Otology*.

[24] Cejas Mendez D. L., de Serdio Arias J. L., Goralsky Filonov S. (1992). Choristoma of the salivary gland and dermoid cyst of the middle ear in a 3-year-old girl. Apropos of a case. *An Otorrinolaringol Ibero Am*.

[25] Munster H. (1994). Salivary gland choristoma in the middle ear (in Danish). *Ugeskrift for Læger*.

[26] Hinni M. L., Beatty C. W. (1996). Salivary gland choristoma of the middle ear: report of a case and review of the literature. *Ear, Nose & Throat Journal*.

[27] Anderhuber W., Beham A., Walch C., Stammberger H. (1996). Choristoma of the middle ear. *European Archives of Oto-Rhino-Laryngology*.

[28] Perry B. P., Scher R. L., Gray L., Bossen E. H., Tucci D. L. (1998). Pathologic quiz case 1. *Archives of Otolaryngology-Head & Neck Surgery*.

[29] Supiyaphun P., Snidvongs K., Shuangshoti S. (2000). Salivary gland choristoma of the middle ear: case treated with KTP laser. *The Journal of Laryngology & Otology*.

[30] Ha S. L., Shin J.-E., Yoon T. H. (2000). Salivary gland choristoma of the middle ear: a case report. *American Journal of Otolaryngology*.

[31] Simoni P., Wiatrak B. J., Kelly D. R. (2003). Choristomatous polyps of the aural and pharyngeal regions: first simultaneous case. *International Journal of Pediatric Otorhinolaryngology*.

[32] Enoz M., Suoglu Y. (2006). Salivary gland choristoma of the middle ear. *The Laryngoscope*.

[33] Lee D. K., Kim J. H., Cho Y. S., Chung W. H., Hong S. H. (2005). Salivary gland choristoma of the middle ear in an infant: a case report. *International Journal of Pediatric Otorhinolaryngology*.

[34] Boleas-Aguirre M. S., Ernst S., Cervera-Paz F. J., Panizo A., Manrique M. (2006). *Revue de Laryngologie—Otologie—Rhinologie*.

[35] Nassar M., Mansour O. (2006). Salivary gland choristoma of the middle ear: a case report and review of the literature. *The Mediterranean Journal of Otology*.

[36] Toros S. Z., Egeli E., Kılıçarslan Y., Gümrükçü G., Gökçeer T., Noşeri H. (2010). Salivary gland choristoma of the middle ear in a child with situs inversus totalis. *Auris Nasus Larynx*.

[37] Amrhein P., Sittel C., Spaich C. (2014). Speicheldrüsenchoristom im Mittelohr bei mittels Array-CGH diagnostiziertem branchiootorenalem Syndrom. *HNO*.

[38] Fois P., Giannuzzi A. L., Paties C. T., Falcioni M. (2014). Salivary gland choristoma of the middle ear. *Ear, Nose & Throat Journal*.

[39] Chen S., Li Y. (2015). Salivary gland choristoma of the middle ear. *Ear, Nose & Throat Journal*.

[40] Noda M., Sugimoto H., Ito M., Yoshizaki T. (2016). Salivary gland choristoma of the middle ear with alopecia. *International Journal of Pediatric Otorhinolaryngology Extra*.

[41] Ziari K., Alizadeh K. (2017). Middle ear salivary gland choristoma: a case report. *Iranian Journal of Pathology*.

[42] Su Q.-Y., Hao S.-J., Wang L., Ye F.-L. (2019). A rare case of salivary gland choristoma in the middle ear with pharyngeal hamartoma. *Chinese Medical Journal*.

[43] Purnell P. R., Interval E., Williams H. J., Cassis A. (2019). Middle ear choristoma presenting as cholesteatoma with conductive hearing loss. *Journal of Surgical Case Reports*.

[44] Dadaş B., Alkan S., Turgut S., Başak T. (2001). Primary papillary adenocarcinoma confined to the middle ear and mastoid. *European Archives of Oto-Rhino-Laryngology*.

[45] Ookouchi Y., Honda N., Gyo K. (2003). Salivary gland choristoma of the middle ear in a child: a case report. *Otolaryngology-Head and Neck Surgery*.

[46] Gómez S. S., Maza Solano J. M., Armas Padrón J. R., Sánchez F. R. (2013). Salivary gland choristoma of the middle ear and review of the literature. *International Journal of Otolaryngology and Head & Neck Surgery*.

[47] Herrero Salado D., Ataman T. (1975). A case of salivary gland choristoma of the middle ear. *The Journal of Laryngology and Otology*.

[48] Yacizi D., Cetik F. (2006). An infrequent mass of the middle ear. *Archives of Otolaryngology–Head & Neck Surgery*.

